# Detection of cancer cells using SapC-DOPS nanovesicles

**DOI:** 10.1186/s12943-016-0519-1

**Published:** 2016-05-10

**Authors:** Harold W. Davis, Nida Hussain, Xiaoyang Qi

**Affiliations:** Division of Hematology/Oncology, Translational Medicine Laboratory, Department of Internal Medicine, University of Cincinnati College of Medicine, and Brain Tumor Center at UC Neuroscience Institute, 3512 Eden Avenue, Cincinnati, OH 45267-0508 USA; Division of Human Genetics, Department of Pediatrics, Cincinnati Children’s Hospital Medical Center, Cincinnati, OH USA

**Keywords:** Phosphatidylserine, Cancer, Tumor imaging, Contrast agents, Saposin C, SapC-DOPS

## Abstract

Unlike normal cells, cancer cells express high levels of phosphatidylserine on the extracellular leaflet of their cell membrane. Exploiting this characteristic, our lab developed a therapeutic agent that consists of the fusogenic protein, saposin C (SapC) which is embedded in dioleoylphosphatidylserine (DOPS) vesicles. These nanovesicles selectively target cancer cells and induce apoptosis. Here we review the data supporting use of SapC-DOPS to locate tumors for surgical resection or for treatment. In addition, there is important evidence suggesting that SapC-DOPS may also prove to be an effective novel cancer therapeutic reagent. Given that SapC-DOPS is easily labeled with lipophilic dyes, it has been combined with the far-red fluorescent dye, CellVue Maroon (CVM), for tumor targeting studies. We also have used contrast agents incorporated in the SapC-DOPS nanovesicles for computed tomography and magnetic resonance imaging, and review that data here. Administered intravenously, the fluorescently labeled SapC-DOPS traversed the blood–brain tumor barrier enabling identification of brain tumors. SapC-DOPS-CVM also detected a variety of other mouse tumors in vivo, rendering them observable by optical imaging using IVIS and multi-angle rotational optical imaging. Dye is detected within 30 min and remains within tumor for at least 7 days, whereas non-tumor tissues were unstained (some dye observed in the liver was transient, likely representing degradation products). Additionally, labeled SapC-DOPS ex vivo delineated tumors in human histological specimens. SapC-DOPS can also be labeled with contrast reagents for computed tomography or magnetic resonance imaging. In conclusion, labeled SapC-DOPS provides a convenient, specific, and nontoxic method for detecting tumors while concurrently offering a therapeutic benefit.

## Background

### Phosphatidylserine and cancer

Phospholipids are arranged asymmetrically in cell membranes, with neutral phospholipids on the outer leaflet and anionic phospholipids, such as phosphatidylethanolamine and phosphatidylserine (PS), located primarily on the inner leaflet of the membrane [1–3]. A translocase, the flippase complex, selective for PE and PS, is an ATP-dependent pump that catalyzes rapid inward migration of these phospholipids [1] to maintain this configuration. Collapse of this asymmetry is an early process in apoptosis that results in the translocation of PS to the exterior of the cell. In a normal cell undergoing apoptosis, the externalized PS serves as a trigger for phagocytes, particularly macrophages, to engulf the cell, minimizing inflammation [4, 5].

Although cancer cells and their associated tumor vasculature also exhibit a high level of PS on the outer leaflet [6–9], this externalized phospholipid is not associated with apoptosis. Critically, the mechanisms by which cancer cells actually resist phagocytosis remain incompletely understood [9]. Compared with non-malignant cells, expression of PS on the cell surface is a consistent marker of malignancy in both primary and metastatic cell lines [6–12]. In their study focused on difficult-to-treat primary cancers, including metastatic melanoma, glioblastoma, and metastatic lesions, Riedl et al. [11] demonstrated the specificity of abundant externalized PS for malignant tumors. While virtually all cancer cells exhibit high external PS compared with normal cells, the quantity of surface PS varies widely among different cancer cells, even of the same type [10, 12]. The increase in surface PS has led to the use of a number of proteins or peptides that bind to PS to study apoptotic and tumor cells [2, 4, 13]; among these are annexin A5 (ANXA5), an endogenous anticoagulant protein, and lactadherin (MFGE8) [2, 14], a major glycoprotein in milk that promotes cellular adhesion. Additionally, monoclonal antibodies to PS have been generated that demonstrate anti-tumor activity [6, 15]. All of these have been conjugated to a number of markers to detect the location of PS.

In this review, we discuss Saposin C-Dioleoylphosphatidylserine (SapC-DOPS), a stable nanovesicle that specifically binds PS but, importantly, also has demonstrated therapeutic properties against a variety of cancer types. Given the specificity of SapC-DOPS for cells that have undergone neoplastic transformation and the resultant enhancement seen on imaging studies owing to the externalization of PS on cancer cells, we review this new paradigm for improved diagnosis and early detection of malignancy that may overcome some of the limitations of current imaging related to the cancer’s type and site or to other underlying medical conditions (e.g., diabetes and kidney disease).

### SapC-DOPS

**Fig. 1:**
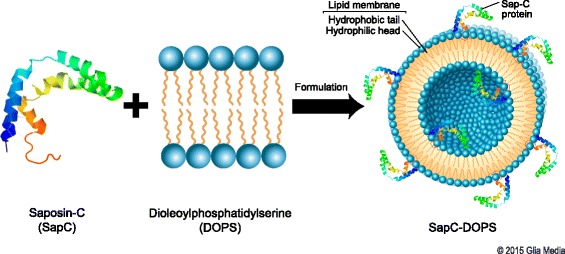
Generation of SapC-DOPS. Saposin C (SapC) is a low molecular weight, heat-stable protein which can fuse lipid vesicles into cells by binding to phosphatidylserine (PS) in an acidic environment. Mixing SapC with dioleoylphosphatidylserine (DOPS) at a low pH results in the formation of SapC-DOPS vesicles with a mean diameter of ~200 nm. Used with permission © 2015 Glia Media

**Fig. 2:**
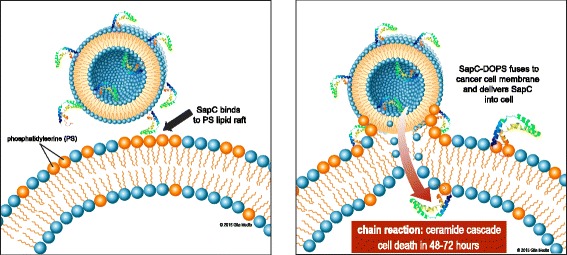
Mechanism of cancer cell killing by SapC-DOPS. SapC-DOPS binds to PS-rich patches of cell membranes. Once SapC-DOPS binds, SapC activates acid sphingomyelinase to initiate the ceramide cascade, which results in cell death. Used with permission © 2015 Glia Media

Saposin C (SapC) is a small, fusogenic glycoprotein that is remarkably heat-stable and protease-resistant [16–19]. While SapC itself is non-enzymatic, it is an activator of lysosomal enzymes, particularly acid sphingomyelinase and acid beta-glucosidase, which catalyze the breakdown of sphingomyelin and glucosylceramide into phosphocholine and ceramide, and glucose and ceramide, respectively [20–22]. Although the precise mechanism is unclear, this increase in ceramide levels may result in cell death, as ceramide has been previously implicated in apoptosis [23], possibly through the actions of caspases [24]. In order for SapC to activate these enzymes, it must bind the PS of the intracellular vesicles’ membranes. In vitro, at low pH, SapC and dioleoylphosphatidylserine (DOPS) will spontaneously form nanovesicles with a mean diameter of approximately 200 nm (Fig. 1). The amino- and carboxyl termini of SapC are amphipathic helices that insert into the lipid bilayer, while the middle region is exposed to solvent. Conformational changes of SapC induced by PS interaction suggest a reorientation of the functional helical domains [25]. Importantly, the cytotoxicity of SapC-DOPS positively correlates with the level of surface PS: the higher the external PS, the more effectively SapC-DOPS [10, 12] will bind the cell and trigger the ceramide cascade, ultimately resulting in apoptosis (Fig. 2)_._

## Main Text

### Use of SapC-DOPS as a tumor detection agent

#### Histological sections

In our lab, the role of PS as a tumor marker has been validated repeatedly for a variety of cancer types [10, 24, 26–30]. In particular, the targeting of PS by SapC-DOPS was demonstrated to be a novel method to achieve accurate and effective identification of cancer cells. The validation of PS as a reliable tumor marker, coupled with the high affinity of SapC-DOPS for PS on a variety of cancer cell surfaces, provides a promising advancement for accurate detection of several cancer types.

**Fig. 3:**
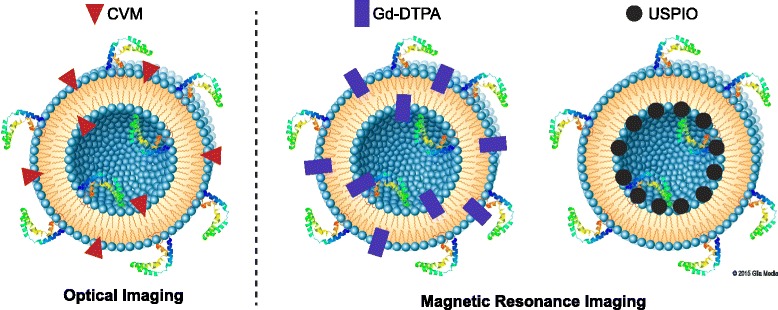
Tumor detection by SapC-DOPS. SapC-DOPS nanovesicles can be labeled with imaging agents during formulation. For histological specimens and in vivo studies with small animals, optical imaging with the far red fluorophore, CellVue Maroon (CVM) can be used. For in vivo MRI imaging, the gadolinium chelate (Gd-DTAP-BSA) or the ultra-small cuperparamagnetic firon oxide (USPIO) can be incorporated and used as MRI contrast agents. Used with permission © 2015 Glia Media

The lipophilic properties of SapC-DOPS make it an ideal carrier of detection moieties, such as fluorescent markers or clinically applicable contrast agents (Fig. 3). Experiments coupling SapC-DOPS to the far-red fluorescent probe CellVue Maroon (CVM) have been performed to distinguish neoplastic tumor regions on histologic slides and individual patient-derived neoplastic cell lines, as well as in vivo [10, 24, 26–28, 31]. In several studies showing the specificity of this reagent, SapC-DOPS-CVM was internalized by live tumor cells but not normal cells, clearly delineating the tumor in tissue slices. A caveat of using histological slides is that their generation requires slicing the cells, so that SapC-DOPS or other PS detection agents will bind to PS on both the exterior and interior of the membrane. However, fluorescently labeled SapC-DOPS can be used with flow cytometry to distinguish only the externalized PS.

Of particular interest is the role of SapC-DOPS in detecting cancers that pose diagnostic challenges because of the tumor site or the intrinsic properties of the malignancy itself, by targeting surface PS of cancer cells and tumor vessels. Such cancers include glioblastoma and pancreatic adenocarcinoma, each of which carries a devastating prognosis despite years of diagnostic and therapeutic research.

#### Fluorescent SapC-DOPS detection in mice

Previous studies have taken in vitro data of fluorescently labeled SapC-DOPS a step further by evaluating diagnostic efficacy in vivo using mouse models. In one such study, mice bearing human neuroblastoma xenografts were injected intravenously with four different reagents: (1) SapC-DOPS conjugated to CVM, (2) unconjugated SapC and CVM, (3) DOPS and CVM, or (4) PBS alone [24]. The mice then underwent optical imaging at time intervals ranging from 0 to 48 h post-injection. The SapC-DOPS-CVM fluorescent signal was diffuse between 0 and 5 h, and had accumulated specifically within the tumor region by 24 h, persisting for up to 100 h. However, there was no fluorescence signal when uncoupled SapC was administered with DOPS-CVM [24]. Similar results were demonstrated by Kaimal et al. [29] in their mouse xenograft models of pancreatic adenocarcinoma and neuroblastoma and a murine rhabdomyosarcoma model. These studies demonstrated the specificity of SapC-DOPS for cells that have undergone neoplastic transformation, with the consequent externalization of PS. As such, this is a new paradigm for improved diagnosis and early detection of malignancy.

In multi-angle rotational optical imaging (MAROI) to detect SapC-DOPS-CVM in mice [32], we used a rotational bed to obtain the in vivo image. Analysis of the MAROI signal curve provided multispectral and multimodal data derived from complete rotational coverage. We confirmed that optimal imaging depended on correct orientation during positioning; the fluorescence intensity decreased by as much as 9–12 % with each 10°of movement. Use of anatomical landmarks and concurrent X-ray imaging achieved both in vivo localization of the tumor and quantitation of fluorescent marker intensity. These findings can then be used in longitudinal studies to correlate fluorescent signal distribution directly with mapping of tumor location.

#### Impediments to clinical detection of malignancy using mulimodality imaging

Among the factors considered when determining an optimal treatment plan for patients diagnosed with cancer, especially important are the size and location of the primary tumor and the extent of metastatic disease, if present. To define these characteristics and facilitate treatment, effective imaging modalities currently available include computed tomography (CT), positron emission tomography (PET), and magnetic resonance imaging (MRI). Each modality can help define cancer staging and diagnosis, but can also be limited in its effectiveness, depending on the type and site of the cancer or the patient’s underlying medical conditions. When such limitations exist, invasive procedures or a combination of diverse imaging techniques may be needed to make a definitive diagnosis; this can be both time-consuming and cost prohibitive (see Table 1).

**Table 1:** Comparison of non-invasive imaging procedures for cancer detection

Imaging method	Benefits	Limitations
Optical Imaging	Fluorescently labeled probes may be sensitive and specific	Limited depth, may be too specific, may require a visit to inject the probe with a follow-up visit to detect where it binds
Computed Tomography (CT)	Fast, highly detailed	Exposure to ionizing radiation, may not be able to differentiate tumor from other lesions, may not detect small tumors, may require potentially toxic contrast agents, not ideal modality for brain tumor detection
Magnetic Resonance Imaging (MRI)	Used for detection of brain tumors, no radiation exposure for patient	Prolonged acquisition time, metal implants preclude this technique, requires potentially toxic contrast agents
Positron Emission Tomography (PET)	Better than CT or MRI for nodal or distant metastases	Poor special resolution, inferior for detection of primary tumors, cannot detect brain tumors, use of FDG impacted in diabetics, exposure to ionizing radiation

CT has played a pivotal role in both diagnosis and staging of malignancy for several years. Although CT has been successful in upstaging many cancers at the time of diagnosis to improve treatment outcomes, several limitations prevent its universal applicability in cancer diagnosis, particularly in cases of local, microscopic disease spread. In a study of 957 lymph nodes evaluated from patients with head and neck cancers, Don et al. [33] found that 20 % of malignant lymph nodes had extracapsular spread; almost one third of these nodes were smaller than 10 mm, which is the size cut-off used to define pathological adenopathy on radiographic review. In addition, central necrosis, a common characteristic used to identify malignant lymph nodes by CT, were found primarily in lymph nodes that were 20 mm or larger, suggesting that central necrosis is a late event in metastatic adenopathy. Such findings suggest a deficiency in our ability to detect metastatic disease early in its course [33].

In contrast with CT and MRI, detection by PET imaging has proven superior for regional nodal and distant metastases, but inferior for several primary malignancies [34]. Moreover, PET has very poor spatial resolution, limiting accurate biopsy because of poor localization of the potential malignancy. While this problem can be offset in part by combined PET-CT scanning, defects in registration between the signals can still pose problems with tumor localization [34].

As such, currently available imaging modalities present shortcomings in our ability to detect occult spread of malignancy, which inevitably leads to delays in diagnosis of metastatic disease until it has spread more widely. At that point, few treatment options may remain. Such studies highlight the need for sensitive imaging techniques that focus on specifically revealing malignant cells, rather than probing tentative neoplastic properties of lymph nodes that may imply, but are not always specific for, malignancy.

Detection of intracranial neoplasms, such as glioblastoma multiforme, also poses a diagnostic challenge by currently available imaging techniques. PET imaging, for example, relies on underlying tissue metabolism to detect malignancy, given that neoplastic cells have increased metabolic activity compared with normal tissue. Non-malignant brain tissue, however, has metabolic activity comparable to tumors found in areas outside of the brain. Because intracranial neoplasms cannot be accurately identified on PET imaging, a second mode of imaging is needed to detect either primary or metastatic disease in the brain. Similarly, a CT of the brain can delineate brain lesions but often cannot definitively distinguish neoplastic tumors from other causes of brain lesions, including infections or demyelinating diseases. Therefore, detection of intracranial malignancy currently relies on MRI for evaluation and characterization. Such studies, however, require prolonged acquisition time, which can cause increased motion artifact and result in poor image quality unless the patient is sedated, as for pediatric patients, which increases patient risk.

Beyond accurate detection of malignancy, limitations in current imaging modalities also relate to any underlying medical conditions of the patient. For example, CT imaging often requires an intravenous contrast agent. Contrast can be particularly nephrotoxic for patients with acute or chronic kidney disease. This comorbidity is common in patients at the time of cancer diagnosis because of poor oral intake and prolonged cachexia, and can also be a frequent consequence of several chemotherapeutic regimens. Thus, contrast-enhanced CT imaging is commonly avoided in patients with underlying kidney disease. Similar caution must be taken when using gadolinium contrast for MRI in patients with kidney disease because of the possibility of adverse outcomes, such as nephrogenic systemic sclerosis, which carries a high rate of morbidity and mortality. Limitations in PET imaging occur in diabetic patients, because blood glucose levels can significantly impact tumor uptake of fluorodeoxyglucose (FDG), the agent used to detect malignancy. In these cases, FDG and glucose compete for glucose transport and phosphorylation [35]. Guidelines currently require both tight glycemic control (i.e., glucose levels below 200 mg/dL) before PET imaging and that patients abstain from all glucose-containing food and drink for at least 6 h prior to the study. However, these restrictions have often proven difficult to achieve, thus compromising imaging quality and accuracy. Advances in combined PET/CT [36] and PET/MRI [37] have solved some of these problems. However, the toxicity of contrast agents and their inability to cross the blood–brain barrier limit their effectiveness and demonstrate the necessity for a diagnostic agent with limited side effects, few clinical restrictions, and enhanced specificity for neoplastic cells.

#### Detection of malignancy using SapC-DOPS in preclinical studies

**Fig. 4:**
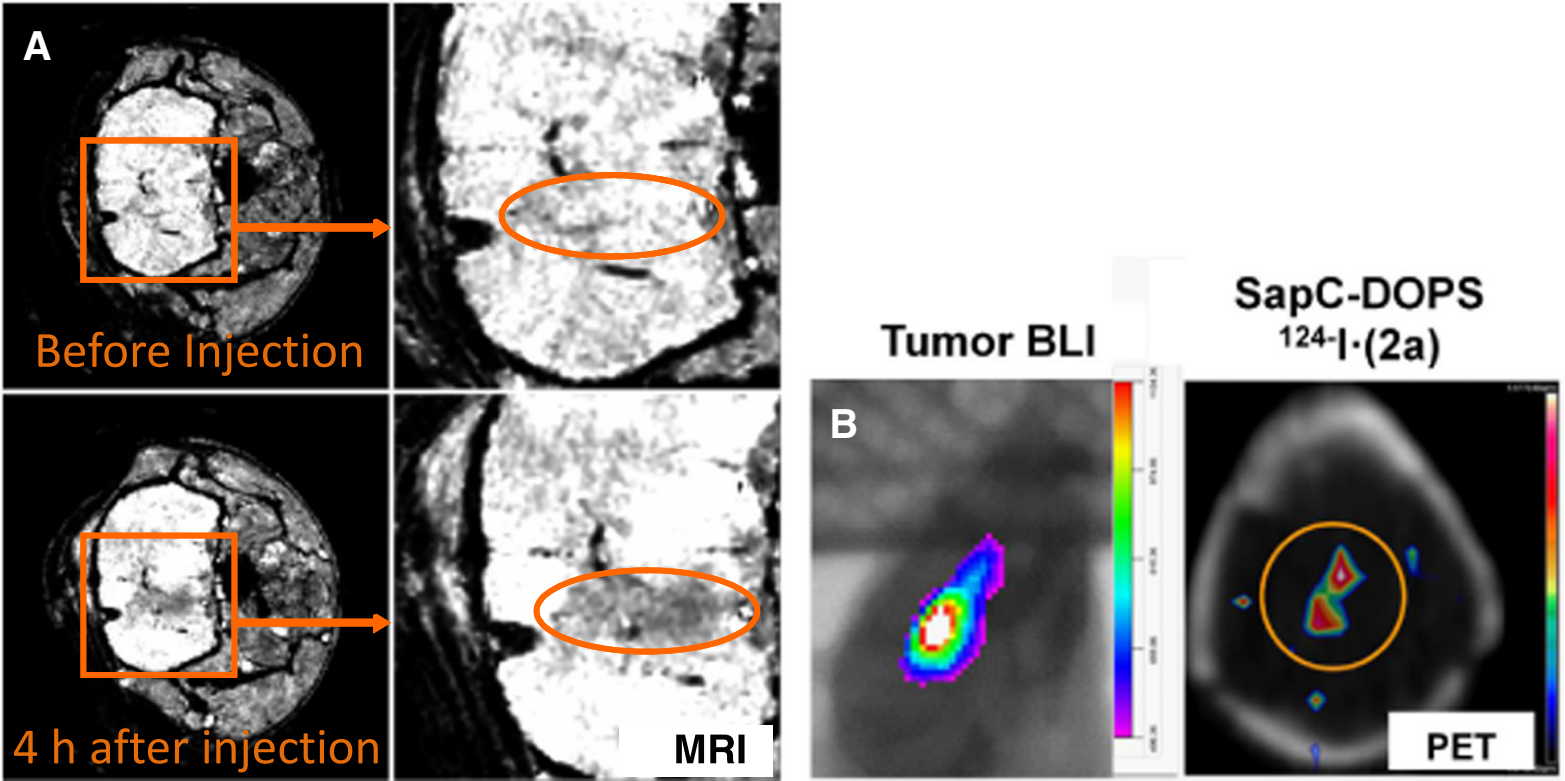
**a** High-resolution MRI of a glioma in a mouse in vivo. MRI was performed at 7T. T2* weighted 3D FLASH sequence (TE/TR = 10 ms/20 ms/FA = 10°) were used with a 320 × 320 × 64 matrix and 3.2 × 3.2 × 0.64 cm FOV resulting in an isotropic 100 mm resolution. Negative contrast enhancement is observed 4 h following SapC-DOPS-USPIO (adapted from [28]). **b** MicroPET imaging of a glioblastoma in a mouse brain 24 h after administration of SapC-DOPS-^124^I · (2a) nanovesicles. 2a is a phenol-substituted analog of indodicarbocyanine (DiD). A CT scan was acquired for anatomical co-registration and attenuation correction of the PET data. Concurrent bioluminescence imaging (BLI) confirmed the presence of glioblastoma (adapted from [40]).

To improve MRI sensitivity, shorten scanning time, and improve safety, we have used SapC-DOPS as a carrier for contrast agents. The method of Bogdanov et al. [38] was implemented to encapsulate ferumoxtran-10, an ultra-small super-paramagnetic iron oxide (USPIO) contrast agent, into SapC-DOPS vesicles. The resulting SapC-DOPS-USPIO was used with MRI to detect tumors in mice [29]. The *T*_*2*_ relaxation time (i.e., time for the transverse magnetization to fall to approximately 37 % of its initial value after magnetization) of subcutaneous xenografts of neuroblastomas or pancreatic tumors was decreased by SapC-DOPS-USPIO, thus indicating the uptake of the agent by tumors. This allowed specific detection of the malignancy (Fig. 4a).

Additionally, we incorporated the paramagnetic contrast agent, gadolinium, into SapC-DOPS vesicles by using the lipophilic gadolinium chelate, gadolinium-DTPA-bis (stearylamide) [39]. These vesicles produced a 9 % increase in the longitudinal relaxation rate (R1) of orthotopic glioblastoma multiforme tumors in mice within 10 h post-injection, but only minimal changes in normal brain tissue; again this demonstrated improved specificity of tumor detection.

We have recently used a phenol-substituted lipophilic dye to label SapC-DOPS with ^124^I, a positron emitter. We then used this labeled SapC-DOPS for PET imaging. As shown in Fig. 4b we were able to selectively enhance the intracranial glioblastomas with PET scanning [40]. Concurrent studies with SapC-DOPS labeled with ^125^I instead of ^124^I indicated that SapC-DOPS specifically targeted the tumor, although some label was detected in the liver and spleen, likely disposal routes.

These studies indicate the ability of SapC-DOPS to transverse the blood brain barrier without requiring either alteration of the barrier or direct intracranial administration of the agent [12]. This suggests a potential role for SapC-DOPS in improving the safety and convenience of detecting (and treating) intracranial neoplasms.

The pharmacologic safety of SapC-DOPS has been evaluated in mice at 12× the typical therapeutic dose of 4 mg/kg of Sap C and 2 mg/kg of DOPS [24]. Even at these levels, no acute toxicity or weight loss was demonstrated with administration. Furthermore, histological examination of vital organs (i.e., lung, liver, spleen, kidney, heart, brain) revealed neither damage nor toxic changes. Chronic toxicity studies were also performed with injection of 2× therapeutic concentrations of SapC-DOPS weekly for 5 weeks. Again, these studies demonstrated no significant toxicity on histological review of the vital organs listed above [24]. In comparison, the contrast agents currently used for image enhancement during CT and MRI scanning can place patients at risk for kidney damage or systemic disease. Beyond the pharmacologic safety evidence, we compared survival data for mice with pancreatic tumors treated with SapC-DOPS versus control groups. Mice treated with SapC-DOPS lived significantly longer compared with untreated control groups: specifically, all control mice had died by 23 weeks after treatment, while 4 of the 6 SapC-DOPS-treated mice were still alive [10]. Similar results were obtained with a brain tumor model [28]. Mice bearing orthotopic glioblastoma multiforme that were treated with DOPS alone all died within 20 days. However, 25 % of those treated with SapC-DOPS survived at least 350 days. In addition, the tumors were smaller in the SapC-DOPS treated mice. Again, these studies provide pre-clinical data to support the safety of systemic SapC-DOPS administration for diagnostic and therapeutic purposes. Although there is some therapeutic value of SapC-DOPS with the dose that would be injected for diagnostic purposes, multiple doses are generally given to shrink tumors. Additionally, as SapC-DOPS is a nanovesicle it can be loaded with radioisotopes or chemotherapeutic drugs to provide further benefit.

## Conclusions

Our data indicate that the novel and PS-targeted nanovesicle, SapC-DOPS, can be used for exposure of hard to detect malignancies, whether due to size or location. Although further studies are required, our preclinical studies suggest that the tumor–selective nanovesicles may greatly contribute to improving the precision of early cancer diagnosis. In addition, SapC-DOPS may have a therapeutic benefit and be used as a “theranostic” compound. Ongoing studies are to provide support for the inclusion of SapC-DOPS in the battery of tests conducted by oncologists to enhance the accuracy and sensitivity of tumor diagnosis.

## Ethics approval

The ethical approval for the animal studies described in this review are provided in the original publications.

## Abbreviations

CT: computed tomography
CVM: CellVue Maroon
FDG: fluorodeoxyglucose
MAROI: multi-angle rotational optical imaging
MRI: magnetic resonance imaging
PET: positron emission tomography
PS: phosphatidylserine
SapC-DOPS: saposin C-Dioleophosphatidylserine
USPIO: ultra-small super-paramagnetic iron oxide
